# Oscillator Strengths for Lines of Ionized Uranium (U II)

**DOI:** 10.6028/jres.080A.045

**Published:** 1976-06-01

**Authors:** Charles H. Corliss

**Affiliations:** Institute for Basic Standards, National Bureau of Standards, Washington, D.C. 20234

**Keywords:** Oscillator strengths for U II, transition probabilities for U II, uranium spectrum

## Abstract

Oscillator strengths for 49 lines of U II recently measured by Voight can be used to calibrate the intensity scale of the U II lines in the NBS Tables of Spectral-Line Intensities and derive a larger set of oscillator strengths of lower precision but consistent with the new measurements. The standard deviation of the differences between the two sets of *gf*-values for the 49 lines is 29 percent. Oscillator strengths of that precision are given for 776 additional lines from the NBS Intensity Tables. The uncertainty in absolute value is 67 percent.

## 1. Introduction

Recently Voigt [1975]^1^ measured oscillator strengths for 49 lines of U II in a wall-stabilized argon arc. His analysis of errors leads to the conclusion that, on a relative scale, his errors do not exceed 10 percent. In his table III of results for U II lines he compares his values with *gf*-values from Corliss and Bozman [1962]. The well-known energy dependent error of their values is clearly exhibited in this table. Nearly all the old values for lines originating from levels above 25 000 cm^−1^ are larger than Voigt’s values, while for lines originating from levels below 25 000 cm^−1^ the old values are nearly all smaller. A new calibration of the level populations in the copper arc of Meggers, Corliss, and Scribner [1975] is clearly required.

## 2. Comparison of Intensities with Oscillator Strengths

We compare the intensities from Meggers, Corliss, and Scribner with the new oscillator strengths by the usual population plot of log *I*λ^3^/*gf_v_* versus upper energy level. The standard deviation of the residuals from the least-squares line fitted to the 49 points was 0.20 dex (±58%). Since Voigt’s *gf*-values have a relative error of only 10 percent, we may interpret the residuals as mostly error in the intensities.

**Figure 1:**
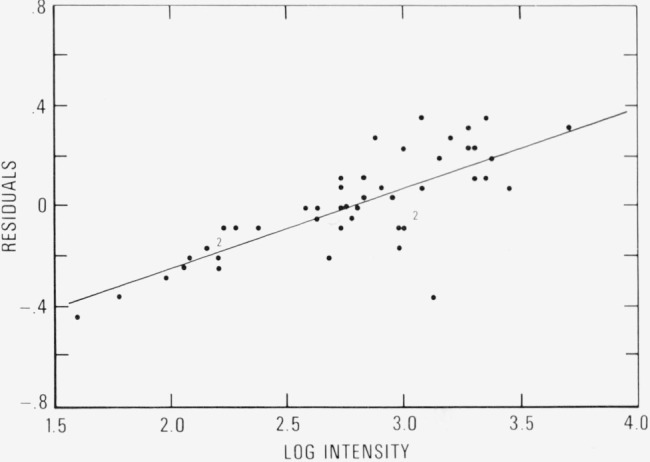
Residuals from a plot of log *Iλ*^3^/gf_v_ versus energy level plotted versus log *I*

The errors in the intensities may be random, systematic or both. Random errors cannot be removed but systematic errors can be removed if they can be specified. There seem to be two possibilities of systematic error in the intensities, i.e., as a function of wavelength or of intensity. The residuals from the plot were plotted against both quantities and there was correlation in each case. It is not surprising that if one quantity were to show correlation the other would also, since atomic spectra usually show a correlation between wavelength and intensity. However, in this case the correlation of the residuals with intensity was better than with wavelength. This correlation plot and its least-squares fitted line are shown in figure 1. The line can be represented by the equation *R = −* 0.894 + 0.323 log *I*. The only badly outlying point represents the spectrum line at 2941 Å.

The intensity correlation shown above implies either that the intensity scale of Meggers, Corliss, and Scribner is too expanded or that Voigt’s scale is too compressed.^2^

## 3. Derivation of New Values

**Figure 2:**
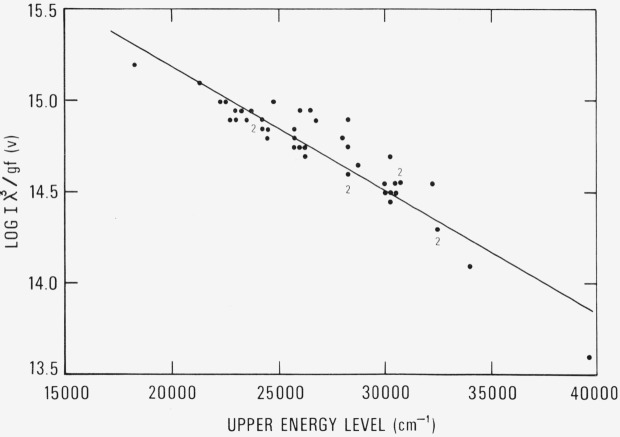
Population plot for the copper arc with the intensities adjusted according to the line in figure 1

Figure 1 clearly demonstrates a systematic discrepancy between the intensity scale of U II lines in Meggers, Corliss, and Scribner and Voigt’s *gf*-values. To derive from our intensities *gf*-values consistent with Voigt’s we should remove this systematic effect. By subtracting *R* from log *I* we obtain a corrected intensity which has the systematic error removed. Log *I*λ^3^/*gf_v_* is then recomputed using the corrected intensity and plotted in figure 2. The standard deviation of the residuals is now reduced to 0.11 dex (±29%), which in fact represents also the standard deviation of the differences between Voigt’s *gf*-values and *gf*-values derived from the corrected intensity scale of U II lines in the Tables of Spectral-Line Intensities.

The equation of the least-squares fitted line in figure 2 is log *I*λ^3^/*gf* = 16.531−0.00006717 *E.* With this equation we calculate *gf* for the 49 lines measured by Voigt and for 776 others.

## 4. Results

The results are given in the tables. In table 1 we give log *gf* as measured by Voigt and as calculated from the Tables of Spectral-Line Intensities. The differences for the 49 lines are given in the fourth column. The standard deviation of the differences is 0.11 dex (±29%). In table 2 we list the wavelength in Angstroms, energy levels in cm^−1^, the weighted transition probability *gA* in units of 10^8^ transitions per second, the weighted oscillator strength, and log *gf* for 825 lines of U II from Meggers, Corliss, and Scribner. Classifications for 680 of these lines are given in the Intensity Tables and 145 more were classified with new levels kindly sent to us by Jean Blaise and Leon Radziemski from the Los Alamos Scientific Laboratory. Earlier references for levels are given in the Intensity Tables.

In a number of cases the intensities in Meggers, Corliss, and Scribner represent the summation of unresolved pairs of lines. When both of the lines originate from the ion, we divided the intensity according to the ratio given in Steinhaus et al. [1972]. The remaining unresolved pairs were not used in this paper.

The error in the relative scale of *gf* as determined above is about 30 percent. To calculate the error in the absolute scale, we add (quadratically) the absolute error of 60 percent determined by Voigt for his absolute scale with which we are calibrated. The uncertainty in our absolute scale is thus about 67 percent. This large absolute error arises from the uncertainty in the continuous background which had to be subtracted from a faint U I line during Voigt’s measurement of the relative intensity of a U I and a U II line in his wall-stabilized arc. A direct measurement of a lifetime in U II would avoid this source of error.

## Figures and Tables

**Table 1:** Comparison of log gf for lines of *U II*

Wavelength Å	Log *gf* (*v*)	Log *gf* (*c*)	Difference
			
2941.92	−0.18	−0.47	−0.29
3111.62	−0.72	−0.86	−0.14
3305.90	−0.92	−0.99	−0.07
3550.82	−1.28	−1.01	0.27
3670.07	−0.59	−0.72	−0.13
3700.58	−1.34	−1.21	0.13
3701.52	−0.62	−0.69	−0.07
3746.41	−1.00	−0.84	0.16
3748.68	−0.64	−0.73	−0.09
3782.84	−1.06	−0.89	0.17
3826.51	−1.39	−1.17	0.22
3831.46	−0.70	−0.59	0.11
3854.66	−0.59	−0.54	0.05
3859.58	−0.62	−0.62	0.00
3865.92	−0.85	−0.77	0.08
3881.46	−1.00	−0.80	0.20
3890.36	−1.00	−0.86	0.14
3932.03	−0.82	−0.89	−0.07
3985.80	−0.72	−0.71	0.01
3992.54	−1.13	−1.02	0.11
4017.72	−0.96	−0.91	0.05
4050.04	−1.11	−0.99	0.12
4051.91	−0.92	−0.95	−0.03
4062.55	−1.12	−1.17	−0.05
4067.76	−1.03	−0.90	0.13
4090.14	−0.70	−0.78	−0.08
4116.10	−1.19	−1.19	0.00
4124.73	−1.26	−1.28	−0.02
4128.34	−1.15	−1.10	0.05
4141.23	−0.70	−0.80	−0.10
4171.59	−0.96	−0.92	0.04
4241.67	−0.68	−0.83	−0.15
4244.37	− 1.28	−1.33	−0.05
4341.69	−1.21	−1.24	−0.03
4372.57	−1.54	−1.63	−0.09
4373.41	−1.62	−1.68	−0.06
4472.34	−1.18	−1.28	−0.10
4515.28	−1.54	−1.56	−0.02
4538.19	−1.55	−1.53	0.02
4543.63	−1.16	−1.24	−0.08
4567.69	−1.64	−1.73	−0.09
4569.91	−1.68	−1.72	−0.04
4570.99	−1.64	−1.68	−0.04
4573.69	−1.48	−1.54	−0.06
4641.65	−1.43	−1.42	0.01
4689.07	−1.68	−1.68	0.00
4722.73	−1.55	−1.58	−0.03
4731.60	−1.48	−1.48	0.00
5492.97	−1.60	−1.70	−0.10

**Table 2:** Oscillator strengths for lines of *U II*

Wavelength Å	Energy levels cm^−1^	gA 10^8/^_S_	gf	log gf
				
2419.57	0–41317	.50	.044	−1.36
2498.83	5526–45533	.41	.038	−1.42
2514.77	4420–44173	.72	.069	−1.16
2549.30	4420–43635	.68	.066	−1.18
2556.19	0–39108	.74	.072	−1.14
2565.41	0–38968	1.0	.099	−1.00
2568.98	5259–44173	.37	.036	−1.44
2569.71	0–38903	.84	.083	−1.08
2584.42	0–38681	.42	.043	−1.37
2616.07	0–38213	.33	.034	−1.46
2621.81	0–38130	.25	.025	−1.59
2639.84	0–37869	.38	.040	−1.40
2641.55	5790–43635	.58	.061	−1.21
2644.12	0–37808	.38	.040	−1.40
2645.47	0–37789	.90	.095	−1.02
2647.02	914–38681	.18	.019	−1.73
2659.02	0–37596	.23	.024	−1.61
2660.14	289–37869	.39	.041	−1.39
2665.87	289–37789	.32	.034	−1.47
2675.88	1749–39108	.47	.050	−1.30
2676.41	6283–43635	1.4	.15	−.84
2683.28	8276–45533	3.0	.33	−.48
2685.98	1749–38968	.76	.082	−1.09
2691.80	8394–45533	1.1	.12	−.94
2705.19	914–37869	.52	.058	−1.24
2709.51	4420–41317	.56	.062	−1.21
2711.10	914–37789	.24	.027	−1.57
2715.54	2295–39108	.40	.044	−1.36
2725.94	2294–38968	.47	.052	−1.28
2739.39	289–36782	.40	.045	−1.34
2746.16	1749–38152	.42	.047	−1.33
2750.13	0–36351	.17	.019	−1.73
2754.16	914–37212	.86	.098	−1.01
2766.88	0–36131	.40	.045	−1.34
2776.29	4585–40594	.46	.053	−1.27
2778.45	9553–45533	.91	.11	−.98
2780.04	0–35960	.39	.045	−1.35
2784.67	289–36189	.37	.043	−1.36
2784.92	8276–44173	.74	.086	−1.07
2793.94	289–36070	1.1	.13	−.90
2797.30	0–35738	.22	.026	−1.59
2802.56	289–35960	1.1	.13	−.89
2805.24	914–36551	.40	.047	−1.33
2810.35	2294–37867	.39	.046	−1.34
2811.38	1749–37308	.64	.076	−1.12
2813.04	4706–40244	.71	.084	−1.08
2815.98	289–35790	.35	.042	−1.38
2821.12	914–36351	1.2	.14	−.86
2824.37	289–35684	.61	.073	−1.13
2832.06	5259–40559	2.3	.28	−.55
2834.55	914–36183	.26	.031	−1.51
2839.89	289–35491	.67	.081	−1.09
2845.96	914–36042	.34	.042	−1.38
2846.14	8510–43635	.81	.099	−1.01
2849.98	5259–40337	.49	.060	−1.23
2850.82	5526–40594	.51	.062	−1.21
2852.75	5526–40570	.81	.099	−1.00
2853.57	1749–36782	.45	.055	−1.26
2854.92	1749–36766	.28	.034	−1.46
2860.47	0–34949	.55	.068	−1.17
2862.62	4585–39508	.43	.053	−1.27
2865.68	0–34885	1.0	.13	−.90
2869.37	1749–36589	.44	.054	−1.26
2874.08	1749–36532	.48	.060	−1.22
2875.20	914–35684	.42	.053	−1.28
2886.45	289–34923	.34	.043	−1.37
2887.59	9553–44173	1.2	.15	−.82
2888.26	0–34612	.55	.069	−1.16
2889.63	289–34885	1.2	.15	−.83
2894.14	5259–39801	1.0	.13	−.88
2898.13	5259–39754	.62	.078	−1.11
2898.71	2294–36782	.39	.049	−1.31
2902.81	0–34439	.20	.025	−1.60
2904.51	289–34708	.39	.049	−1.31
2912.58	289–34612	.28	.036	−1.45
2914.25	289–34593	.47	.060	−1.22
2918.97	5260–39508	.65	.083	−1.08
2923.17	0–34199	.19	.025	−1.60
2925.98	1749–35915	.38	.048	−1.32
2927.38	289–34439	.46	.059	−1.23
2930.59	0–34112	.22	.029	−1.54
2931.89	0–34097	.34	.044	−1.36
2936.45	2294–36339	.55	.071	−1.15
2936.78	1749–35790	.25	.032	−1.49
2937.35	914–34949	.26	.033	−1.48
2939.49	9626–43635	.98	.13	−.89
2941.92	5527–39508	2.6	.34	−.47
2942.12	2294–36273	.48	.062	−1.21
2942.85	915–34885	.44	.057	−1.24
2943.40	4420–38385	.44	.057	−1.25
2945.89	1749–35684	.43	.057	−1.25
2948.09	289–34199	.46	.060	−1.22
2949.61	4420–38313	.65	.085	−1.07
2955.65	289–34112	.36	.048	−1.32
2957.74	4585–38385	.56	.073	−1.14
2959.85	2294–36070	.49	.065	−1.19
2962.78	1749–35491	.45	.059	−1.23
2966.66	914–34612	.39	.052	−1.28
2967.94	289–33972	.65	.086	−1.07
2968.40	914–34593	.31	.041	−1.39
2970.48	6283–39938	.56	.074	−1.13
2976.35	289–33877	.51	.067	−1.17
2980.28	5259–38803	.47	.063	−1.20
2982.74	289–33805	.42	.057	−1.25
2984.61	2295–35790	.81	.11	−.97
2987.80	289–33748	.27	.037	−1.44
2989.88	1749–35185	.49	.066	−1.18
2999.22	2294–35627	.37	.050	−1.30
3000.09	4420–37743	.51	.069	−1.16
3001.21	1749–35059	.27	.036	−1.44
3007.91	5667–38903	1.0	.14	−.86
3010.75	289–33493	.27	.036	−1.44
3012.45	289–33475	.13	.024	−1.61
3012.71	914–34097	.29	.040	−1.40
3013.37	4420–37596	.63	.086	−1.06
3016.96	1749–34885	.47	.065	−1.19
3018.10	5259–38383	.52	.071	−1.15
3020.24	4663–37764	.41	.056	−1.25
3022.21	1749–34827	.80	.11	−.96
3031.99	4663–37635	1.2	.17	−.77
3033.19	1749–34708	.66	.091	−1.04
3035.96	1749–34678	.25	.035	−1.45
3038.05	0–32905	.26	.037	−1.44
3039.14	4663–37558	.54	.075	−1.12
3039.26	5259–38152	.70	.096	−1.02
3040.46	914–33794	.22	.031	−1.51
3043.79	1749–34593	.34	.048	−1.32
3044.16	4663–37504	1.0	.14	−.85
3046.46	289–33104	.27	.038	−1.42
3050.20	0–32775	.55	.077	−1.11
3056.72	289–32994	.27	.038	−1.42
3062.54	1749–34392	.75	.11	−.97
3063.88	2295–34923	.23	.032	−1.49
3066.87	8755–41352	.72	.10	−.99
3068.65	5790–38368	.79	.11	−.95
3075.45	289–32795	.19	.027	−1.56
3081.19	8510–40956	.86	.12	−.91
3084.24	2295–34708	.26	.037	−1.43
3086.70	5401–37789	.36	.051	−1.29
3086.80	11787–44173	.96	.14	−.86
3087.11	2294–34678	.26	.037	−1.43
3088.99	1749–34112	.38	.054	−1.27
3090.36	289–32638	.26	.037	−1.43
3094.83	289–32591	.19	.027	−1.57
3095.04	1749–34049	.43	.061	−1.21
3095.23	2295–34593	.35	.050	−1.30
3095.75	5259–37552	.79	.11	−.95
3098.01	5716–37985	.84	.12	−.92
3103.77	914–33124	.20	.030	−1.53
3105.10	2294–34490	.29	.042	−1.37
3105.65	5526–37716	.42	.060	−1.22
3111.62	1749–33877	.95	.14	−.86
3112.25	289–32410	.29	.043	−1.37
3119.35	5260–37308	1.1	.16	−.81
3121.09	4663–36694	.36	.052	−1.28
3121.33	0–32028	.20	.029	−1.53
3124.43	8347–40344	.86	.13	−.90
3126.70	4663–36637	.45	.066	−1.18
3129.73	7166–39108	.71	.10	−.98
3130.73	289–32221	.15	.023	−1.65
3131.99	5716–37635	.57	.083	−1.08
3133.42	2295–34199	.21	.031	−1.51
3133.92	1749–33648	.26	.038	−1.42
3136.89	6283–38152	.52	.077	−1.11
3138.51	4420–36273	.34	.050	−1.30
3139.60	5716–37558	1.1	.17	−.78
3141.95	2294–34112	.28	.042	−1.38
3144.96	5716–37504	.94	.14	−.86
3145.56	5527–37308	1.0	.15	−.82
3146.75	4663–36433	.40	.060	−1.22
3148.56	0–31751	.20	.029	−1.54
3151.08	1749–33475	.25	.038	−1.42
3153.12	8853–40559	1.8	.27	−.57
3155.41	4663–36346	.40	.059	−1.23
3155.86	2294–33972	.41	.061	−1.21
3160.77	5716–37345	.55	.082	−1.09
3165.28	6283–37867	.37	.056	−1.25
3165.50	2294–33876	.35	.053	−1.28
3170.86	5716–37244	.46	.069	−1.16
3171.37	5260–36782	.43	.065	−1.19
3173.71	0–31499	.14	.021	−1.68
3175.36	8853–40337	.74	.11	−.95
3176.21	1749–33224	.43	.065	−1.19
3177.33	5716–37180	.79	.12	−.92
3179.04	289–31736	.25	.039	−1.41
3179.83	914–32353	.28	.042	−1.37
3180.20	1749–33184	.32	.048	−1.32
3185.71	1749–33130	.36	.055	−1.26
3188.34	1749–33104	.32	.048	−1.32
3190.89	5259–36589	.26	.040	−1.40
3191.76	4663–35985	.24	.036	−1.44
3200.14	5716–36956	.61	.094	−1.03
3206.05	914–32096	.36	.056	−1.25
3206.23	2295–33475	.26	.040	−1.40
3213.09	8394–39508	.41	.064	−1.19
3218.34	5526–36589	.55	.085	−1.07
3219.17	6283–37338	.47	.074	−1.13
3224.26	5526–36532	.58	.090	−1.05
3226.17	914–31902	.28	.044	−1.36
3232.16	289–31219	.51	.080	−1.09
3235.23	8853–39754	.43	.068	−1.17
3238.46	914–31784	.13	.020	−1.70
3240.35	8347–39199	.40	.063	−1.20
3241.99	914–31751	.26	.041	−1.39
3244.79	2295–33104	.25	.039	−1.41
3246.39	289–31083	.24	.037	−1.43
3253.75	289–31014	.18	.029	−1.54
3257.26	8276–38968	.39	.061	−1.21
3261.72	1749–32399	.35	.056	−1.25
3265.81	914–31526	.34	.054	−1.26
3269.78	289–30863	.11	.018	−1.75
3270.12	289–30860	.37	.059	−1.23
3271.45	5401–35960	.24	.039	−1.41
3280.00	914–31393	.19	.031	−1.51
3282.48	0–30455	.17	.027	−1.57
3283.10	914–31364	.22	.036	−1.44
3285.22	1749–32179	.34	.055	−1.26
3288.21	289–30691	.36	.058	−1.24
3294.44	8521–38867	.53	.086	−1.07
3299.70	2294–32591	.30	.049	−1.31
3303.60	289–30550	.27	.043	−1.36
3305.90	0–30240	.62	.10	−.99
3307.55	5526–35751	.24	.039	−1.41
3311.67	1749–31936	.33	.054	−1.27
3313.94	289–30455	.26	.043	−1.36
3318.79	4585–34708	.20	.034	−1.47
3319.32	12549–42666	1.1	.18	−.74
3322.12	4585–34678	.42	.069	−1.16
3325.66	0–30060	.16	.026	−1.58
3327.50	914–30958	.18	.030	−1.52
3329.92	2294–32316	.35	.058	−1.23
3332.42	5791–35790	.24	.040	−1.40
3336.68	5790–35751	.38	.064	−1.19
3337.79	289–30240	.31	.052	−1.28
3338.48	915–30860	.18	.030	−1.52
3341.66	2295–32211	.46	.077	−1.11
3342.68	1749–31656	.32	.053	−1.27
3344.87	5526–35414	.37	.061	−1.21
3354.50	2294–32096	.33	.055	−1.26
3355.11	289–30085	.10	.017	−1.77
3357.93	289–30060	.24	.040	−1.39
3361.73	2294–32032	.22	.037	−1.43
3367.90	0–29683	.13	.022	−1.65
3368.81	6283–35958	.52	.088	−1.05
3370.13	5260–34923	.29	.050	−1.30
3372.01	289–29936	.20	.035	−1.46
3375.78	8853–38468	.77	.13	−.88
3376.55	2294–31902	.18	.031	−1.50
3382.68	2294–31845	.13	.023	−1.64
3384.45	289–29827	.20	.035	−1.46
3386.13	914–30438	.22	.038	−1.42
3392.99	4585–34049	.19	.032	−1.49
3393.91	2294–31751	.33	.057	−1.25
3394.78	5260–34708	.58	.10	−1.00
3395.32	1749–31193	.30	.052	−1.28
3398.26	5259–34678	.33	.057	−1.24
3401.01	289–29683	.24	.041	−1.38
3401.87	4585–33972	.19	.032	−1.49
3403.55	5259–34632	.51	.089	−1.05
3406.27	915–30263	.22	.038	−1.42
3411.53	2294–31598	.32	.056	−1.25
3412.10	8853–38152	.89	.16	−.81
3421.69	8379–37596	.68	.12	−.92
3422.35	5401–34612	.21	.036	−1.44
3423.01	5526–34732	.44	.077	−1.12
3424.56	1749–30941	.47	.082	−1.09
3426.39	11382–40559	.90	.16	−.80
3430.48	6283–35425	.23	.041	−1.38
3431.54	5791–34923	.35	.061	−1.21
3433.71	289–29403	.093	.016	−1.79
3434.15	1749–30860	.28	.049	−1.31
3436.78	4706–33794	.29	.052	−1.29
3451.21	1749–30716	.18	.033	−1.49
3453.57	8853–37801	.99	.18	−.75
3453.78	5667–34612	.33	.060	−1.22
3454.23	5790–34732	.57	.10	−.99
3455.74	1749–30678	.25	.045	−1.35
3457.05	5716–34634	.56	.10	−1.00
3457.71	5526–34439	.54	.097	−1.01
3458.68	2294–31199	.12	.022	−1.66
3472.51	11797–40586	1.6	.29	−.54
3472.56	2295–31083	.19	.035	−1.45
3474.99	914–29683	.16	.028	−1.55
3476.44	8423–37180	.50	.090	−1.04
3477.50	4663–33411	.17	.032	−1.50
3452.49	1749–30455	.49	.089	−1.05
3486.30	7598–36273	.37	.068	−1.17
3459.57	5791–34439	.42	.078	−1.11
3490.24	914–29557	.24	.044	−1.36
3493.33	5259–33877	.57	.11	−.98
3494.84	10198–38803	1.1	.20	−.71
3495.60	4585–33184	.17	.031	−1.51
3495.75	1749–30347	.11	.020	−1.70
3496.42	1749–30341	.41	.075	−1.12
3497.62	8755–37338	.51	.094	−1.03
3499.33	2295–30863	.26	.048	−1.32
3505.07	5259–33781	.50	.091	−1.04
3505.45	0–28518	.13	.024	−1.61
3508.85	1749–30240	.29	.053	−1.28
3509.67	8853–37338	.99	.18	−.74
3511.58	289–28758	.14	.025	−1.60
3513.37	8853–37308	.51	.095	−1.02
3515.24	4420–32860	.22	.041	−1.39
3516.35	5667–34097	.31	.058	−1.24
3517.05	6283–34708	.34	.064	−1.19
3519.96	914–29316	.29	.053	−1.28
3520.79	6283–34678	.40	.075	−1.13
3521.48	5259–33648	.25	.047	−1.33
3523.33	4663–33037	.17	.031	−1.51
3523.57	11382–39754	1.0	.19	−.72
3525.73	9241–37596	.34	.063	−1.20
3526.60	289–28636	.19	.035	−1.46
3528.69	10198–38529	.86	.16	−.80
3529.77	0–28322	.081	.015	−1.82
3531.11	1749–30060	.32	.060	−1.22
3533.57	915–29206	.39	.073	−1.14
3537.45	5259–33520	.29	.054	−1.27
3540.46	5716–33953	.72	.14	−.87
3543.16	5260–33475	.39	.074	−1.13
3544.21	6283–34490	.34	.063	−1.20
3546.68	1749–29936	.23	.043	−1.37
3547.19	1749–29932	.32	.060	−1.22
3550.82	0–28154	.52	.097	−1.01
3551.04	289–28441	.13	.025	−1.60
3552.17	2294–30438	.30	.057	−1.25
3560.44	1749–29827	.16	.031	−1.51
3564.59	8347–36393	.62	.12	−.93
3564.88	9553–37596	.55	.10	−.98
3570.93	4420–32416	.15	.029	−1.53
3571.56	5790–33781	.26	.050	−1.30
3571.69	4420–32410	.25	.047	−1.33
3576.22	10198–38152	.82	.16	−.80
3578.72	8276–36211	1.2	.22	−.65
3581.84	5716–33627	.54	.10	−.99
3590.32	5260–33104	.43	.083	−1.08
3590.50	915–28758	.23	.045	−1.34
3594.95	1749–29557	.25	.045	−1.32
3596.88	1749–29543	.12	.023	−1.63
3599.84	8347–36118	.44	.085	−1.07
3605.48	1749–29476	.14	.026	−1.58
3606.32	4663–32384	.45	.087	−1.06
3608.96	8510–36211	.45	.087	−1.06
3609.68	5716–33411	.40	.078	−1.11
3610.49	6283–33972	.32	.062	−1.21
3611.24	2294–29978	.11	.021	−1.68
3612.67	915–28587	.16	.032	−1.50
3616.76	289–27930	.21	.041	−1.39
3618.49	9553–37181	.52	.10	−.99
3623.06	915–28507	.26	.051	−1.29
3628.35	8521–36074	.27	.054	−1.27
3632.87	10198–37716	.35	.070	−1.15
3635.40	0–27499	.12	.023	−1.64
3640.95	1749–29206	.21	.042	−1.38
3644.85	2294–29722	.078	.016	−1.81
3645.03	914–28341	.22	.043	−1.36
3649.51	4706–32099	.16	.032	−1.49
3659.01	4706–32028	.26	.052	−1.29
3662.33	4663–31961	.24	.049	−1.31
3666.21	8521–35790	.34	.069	−1.16
3670.07	915–28154	.95	.19	−.72
3672.58	8853–36074	.52	.11	−.98
3674.99	8755–35958	.23	.045	−1.32
3675.08	5259–32462	.23	.047	−1.33
3676.56	6283–33475	.43	.087	−1.06
3677.64	11784–38968	.82	.17	−.78
3678.75	8347–35523	.77	.16	−.81
3682.04	5259–32410	.34	.069	−1.16
3691.92	4706–31784	.55	.11	−.95
3693.70	7547–34612	.61	.12	−.91
3697.93	8755–35790	.55	.11	−.94
3700.58	915–27929	.30	.062	−1.21
3701.52	5527–32535	1.0	.20	−.69
3705.04	2294–29277	.18	.036	−1.44
3705.97	10740–37716	.47	.096	−1.02
3707.65	9626–36589	.39	.081	−1.09
3714.76	2295–29206	.21	.043	−1.37
3717.42	8521–35414	.65	.13	−.87
3718.11	1749–28636	.25	.052	−1.28
3718.61	5526–32410	.27	.055	−1.26
3724.98	1749–28587	.17	.036	−1.45
3725.07	5526–32364	.25	.053	−1.28
3725.65	7166–33999	.28	.058	−1.23
3730.13	5790–32591	.23	.047	−1.33
3731.67	5526–32316	.20	.043	−1.37
3732.62	914–27698	.22	.046	−1.34
3737.25	10740–37490	.75	.16	−.80
3738.05	5791–32535	.67	.14	−.85
3744.24	7598–34298	.55	.12	−.94
3746.41	5527–32211	.69	.15	−.84
3747.14	8521–35201	.70	.15	−.83
3745.68	5716–32384	.89	.19	−.73
3752.66	5526–32166	.44	.093	−1.03
3754.31	0–26628	.12	.026	−1.58
3755.45	5790–32410	.46	.097	−1.01
3756.92	4706–31316	.30	.063	−1.20
3759.23	5667–32261	.45	.095	−1.02
3759.88	5259–31848	.20	.043	−1.37
3760.88	0–26581	.14	.031	−1.52
3761.96	914–27489	.13	.027	−1.57
3762.11	1749–28322	.14	.030	−1.52
3764.57	5716–32272	.56	.12	−.92
3768.80	5790–32316	.35	.075	−1.13
3772.82	8347–34545	.44	.094	−1.02
3780.72	915–27357	.22	.048	−1.32
3782.84	289–26716	.60	.13	−.89
3783.84	5791–32211	.51	.11	−.96
3786.57	8521–34923	.33	.071	−1.15
3787.23	5259–31656	.27	.058	−1.23
3790.22	5790–32166	.22	.046	−1.33
3790.33	914–27290	.10	.022	−1.66
3793.10	4585–30941	.51	.11	−.96
3793.57	914–27267	.22	.048	−1.32
3795.13	2294–28636	.19	.041	−1.39
3796.54	4663–30996	.29	.062	−1.21
3796.84	6445–32775	.38	.082	−1.09
3799.20	8394–34708	.51	.11	−.96
3802.27	2294–28587	.20	.043	−1.37
3803.35	0–26285	.087	.019	−1.72
3809.22	5716–31961	.46	.10	−1.00
3813.79	2295–28507	.27	.059	−1.23
3814.07	915–27126	.22	.047	−1.33
3818.46	1749–27930	.11	.025	−1.61
3822.54	4706–30859	.18	.039	−1.41
3826.51	289–26415	.31	.068	−1.17
3829.03	915–27023	.16	.035	−1.46
3829.39	4585–30691	.24	.052	−1.28
3831.46	4663–30756	1.2	.26	−.59
3835.92	7598–33660	.39	.086	−1.07
3838.15	2294–28341	.17	.038	−1.42
3845.12	5526–31526	.20	.044	−1.36
3845.26	7547–33546	.37	.082	−1.09
3845.37	8394–34392	.42	.093	−1.03
3848.60	5259–31235	.49	.11	−.96
3849.71	8522–34490	.31	.070	−1.16
3849.85	0–25967	.12	.026	−1.58
3854.66	4663–30599	1.3	.29	−.54
3859.58	289–26191	1.1	.24	−.62
3864.30	4585–30455	.27	.060	−1.22
3864.48	4663–30533	.27	.061	−1.21
3865.92	2295–28154	.77	.17	−.77
3866.80	914–26768	.21	.047	−1.33
3868.42	4421–30263	.16	.037	−1.43
3870.02	5667–31499	.32	.071	−1.15
3871.88	8347–34167	.26	.058	−1.24
3874.04	289–26094	.26	.059	−1.23
3876.59	9626–35414	.36	.082	−1.09
3878.09	8853–34632	.98	.22	−.66
3881.46	4585–30341	.70	.16	−.80
3882.36	1749–27499	.28	.063	−1.20
3883.28	8423–34167	.66	.15	−.83
3884.68	8755–34490	.51	.11	−.94
3887.70	10198–35913	.52	.12	−.93
3890.36	289–25986	.61	.14	−.86
3891.68	8510–34199	.30	.069	−1.16
3892.41	8755–34439	.50	.11	−.94
3892.68	5260–30941	.56	.13	−.90
3895.27	4421–30085	.23	.052	−1.29
3896.78	4585–30240	.43	.097	−1.01
3897.05	10740–36393	.68	.15	−.81
3897.69	5667–31316	.19	.044	−1.35
3899.07	4421–30060	.22	.050	−1.30
3899.48	4663–30301	.27	.061	−1.22
3899.78	2295–27929	.35	.080	−1.10
3902.56	289–25906	.19	.044	−1.35
3904.30	8347–33953	.73	.17	−.78
3904.56	5260–30863	.29	.066	−1.18
3904.85	8510–34112	.30	.069	−1.16
3908.47	8394–33972	.29	.067	−1.17
3909.06	5790–31364	.20	.045	−1.35
3911.67	4420–29978	.31	.072	−1.14
3914.20	12055–37596	.70	.16	−.79
3915.88	8423–33953	.64	.15	−.83
3918.06	4421–29936	.12	.028	−1.56
3921.55	0–25492	.11	.025	−1.60
3923.05	8394–33877	.29	.067	−1.17
3924.27	4585–30060	.22	.051	−1.29
3927.76	6283–31736	.33	.077	−1.11
3930.98	289–25720	.20	.045	−1.34
3932.03	289–25714	.55	.13	−.89
3933.03	5259–30678	.18	.041	−1.39
3935.38	2294–27698	.29	.067	−1.17
3940.49	914–26285	.18	.042	−1.38
3942.55	8755–34112	.41	.096	−1.02
3944.13	4585–29932	.25	.059	−1.23
3953.58	2294–27581	.21	.048	−1.32
3954.62	5716–30996	.25	.058	−1.24
3954.67	8347–33627	.59	.14	−.86
3962.77	4706–29934	.22	.051	−1.29
3964.67	7166–32382	.37	.088	−1.06
3964.96	0–25213	.093	.022	−1.66
3966.40	2295–27499	.18	.041	−1.38
3966.52	289–25492	.24	.056	−1.25
3969.02	8400–33588	.28	.066	−1.18
3978.80	8394–33520	.38	.090	−1.04
3985.80	5260–30341	.81	.19	−.71
3988.64	8276–33340	.21	.049	−1.31
3988.89	2295–27357	.15	.035	−1.46
3990.42	915–25967	.22	.052	−1.29
3992.54	5716–30756	.40	.095	−1.02
3994.29	289–25317	.079	.019	−1.73
4002.34	4706–29684	.15	.037	−1.43
4003.20	2294–27267	.11	.026	−1.59
4004.06	1749–26716	.20	.049	−1.31
4009.17	6283–31219	.20	.047	−1.32
4011.45	5401–30323	.16	.039	−1.41
4014.16	8755–33660	.22	.053	−1.28
4017.72	5716–30599	.51	.12	−.91
4018.99	289–25163	.14	.035	−1.46
4026.02	2295–27126	.080	.019	−1.71
4031.31	914–25713	.10	.025	−1.60
4033.43	4421–29206	.18	.043	−1.37
4033.73	7598–32382	.32	.079	−1.10
4044.42	5259–29978	.44	.11	−.97
4050.04	0–24684	.42	.10	−.99
4051.91	5260–29932	.45	.11	−.95
4053.03	1749–26415	.12	.031	−1.51
4054.31	6283–30941	.35	.087	−1.06
4058.19	289–24923	.18	.044	−1.36
4060.10	4706–29329	.11	.028	−1.55
4061.74	7598–32211	.22	.054	−1.27
4062.55	0–24608	.28	.068	−1.17
4066.80	8521–33104	.25	.062	−1.20
4067.76	6283–30860	.51	.13	−.90
4071.11	12033–36589	1.1	.26	−.58
4074.49	1749–26285	.17	.043	−1.37
4076.69	914–25437	.16	.040	−1.39
4080.61	12033–36532	.90	.23	−.65
4084.93	5791–30263	.24	.059	−1.23
4088.25	0–24453	.12	.031	−1.51
4090.14	1749–26191	.66	.17	−.78
4091.49	10198–34632	.51	.13	−.89
4094.62	11544–35960	.32	.080	−1.10
4095.75	6283–30691	.28	.070	−1.15
4098.03	289–24684	.18	.046	−1.34
4106.38	1749–26094	.20	.050	−1.30
4106.93	0–24342	.098	.025	−1.60
4113.11	0–24305	.065	.017	−1.78
4116.10	0–24288	.25	.064	−1.19
4124.73	1749–25986	.21	.053	−1.28
4128.34	4420–28636	.31	.080	−1.10
4133.20	5790–29978	.19	.048	−1.32
4135.76	6283–30455	.15	.039	−1.40
4136.81	4421–28587	.15	.038	−1.41
4138.66	9626–33781	.26	.066	−1.18
4139.14	0–24152	.093	.024	−1.62
4141.23	8394–32535	.62	.16	−.80
4144.70	2295–26415	.11	.029	−1.54
4145.39	8521–32638	.15	.038	−1.41
4155.41	6283–30341	.31	.080	−1.10
4163.68	0–24010	.14	.036	−1.44
4164.79	289–24293	.049	.013	−1.90
4165.68	289–24288	.12	.032	−1.50
4171.59	1749–25714	.46	.12	−.92
4172.97	6283–30240	.20	.051	−1.29
4174.19	5527–29476	.24	.062	−1.21
4179.00	4585–28507	.15	.039	−1.41
4184.89	8521–32410	.19	.049	−1.31
4188.07	289–24159	.094	.025	−1.61
4189.28	289–24152	.13	.034	−1.47
4197.52	8394–32211	.32	.083	−1.08
4200.10	6283–30085	.10	.027	−1.57
4204.37	0–23778	.086	.023	−1.64
4210.45	1749–25492	.065	.017	−1.76
4211.66	4585–28322	.21	.056	−1.25
4212.26	4421–28154	.14	.038	−1.42
4214.42	289–24010	.052	.014	−1.86
4227.33	6283–29932	.12	.032	−1.50
4228.76	4706–28347	.10	.027	−1.57
4232.04	289–23911	.047	.013	−1.90
4240.59	5259–28834	.085	.023	−1.64
4241.67	4585–28154	.54	.15	−.83
4244.37	0–23553	.17	.046	−1.33
4252.43	4420–27930	.15	.041	−1.38
4267.30	915–24342	.080	.022	−1.66
4268.85	2294–25713	.068	.019	−1.73
4269.61	1749–25163	.10	.027	−1.56
4273.98	915–24305	.055	.015	−1.82
4276.47	5259–28636	.11	.029	−1.53
4282.03	289–23635	.092	.025	−1.60
4282.46	4585–27929	.15	.042	−1.37
4287.87	0–23315	.088	.024	−1.62
4297.11	289–23553	.075	.021	−1.68
4301.47	0–23241	.061	.017	−1.77
4310.39	6283–29476	.095	.027	−1.58
4313.88	1749–24923	.080	.022	−1.65
4319.78	2294–25437	.041	.012	−1.94
4325.90	5526–28636	.099	.028	−1.56
4341.69	289–23315	.20	.057	−1.24
4347.19	915–23911	.080	.023	−1.64
4362.26	0–22917	.081	.023	−1.64
4362.93	4585–27499	.084	.024	−1.62
4372.57	915–23778	.082	.024	−1.63
4373.41	1749–24608	.073	.021	−1.68
4402.30	9075–31784	.077	.022	−1.65
4402.44	8510–31219	.11	.033	−1.48
4415.24	0–22642	.064	.019	−1.73
4426.68	4706–27290	.082	.024	−1.62
4427.65	289–22868	.056	.017	−1.78
4433.89	8394–30941	.14	.043	−1.37
4434.53	1749–24293	.070	.021	−1.68
4462.97	915–23315	.075	.022	−1.65
4465.13	2295–24684	.055	.017	−1.78
4472.34	289–22642	.17	.052	−1.28
4477.71	915–23241	.044	.013	−1.87
4490.84	1749–24010	.068	.021	−1.69
4510.32	0–22165	.038	.012	−1.94
4515.28	289–22429	.091	.028	−1.56
4538.19	1749–23778	.096	.030	−1.53
4543.63	915–22917	.19	.058	−1.24
4545.58	2295–24288	.089	.028	−1.56
4553.86	915–22868	.029	.0091	−2.04
4555.10	8394–30341	.15	.046	−1.34
4567.69	1749–23635	.059	.019	−1.73
4569.91	289–22165	.061	.019	−1.72
4570.99	6283–28154	.066	.021	−1.68
4573.69	2295–24152	.091	.029	−1.54
4584.85	1749–23553	.041	.013	−1.89
4601.13	915–22642	.035	.011	−1.95
4603.66	2295–24010	.082	.026	−1.58
4605.15	5791–27499	.075	.024	−1.62
4609.86	289–21975	.026	.0082	−2.09
4622.43	5260–26887	.055	.018	−1.75
4627.08	4585–26191	.17	.054	−1.27
4641.65	8394–29932	.12	.038	−1.42
4646.60	915–22429	.088	.029	−1.54
4666.86	289–21710	.058	.019	−1.72
4671.41	4585–25986	.090	.030	−1.53
4689.07	0–21320	.063	.021	−1.68
4700.98	289–21555	.027	.0088	−2.05
4702.05	4706–25967	.057	.019	−1.73
4702.52	2295–23553	.062	.021	−1.68
4722.73	1749–22917	.078	.026	−1.58
4731.60	4585–25714	.099	.033	−1.48
4755.73	0–21021	.043	.014	−1.84
4769.26	0–20961	.035	.012	−1.92
4772.70	2295–23241	.060	.021	−1.69
4779.63	914–21831	.030	.010	−1.98
4819.54	4421–25163	.051	.018	−1.75
4847.65	2295–22917	.048	.017	−1.77
4858.08	4585–25163	.074	.026	−1.58
4859.68	0–20571	.033	.012	−1.93
4861.02	5402–25967	.094	.033	−1.48
4883.78	2294–22764	.033	.012	−1.92
4886.33	5527–25986	.040	.014	−1.85
4899.29	915–21320	.043	.015	−1.81
4913.16	2295–22642	.044	.016	−1.79
4924.64	5667–25967	.051	.019	−1.73
4933.66	4421–24684	.033	.012	−1.92
4950.17	5791–25986	.040	.015	−1.83
4972.10	915–21021	.024	.0089	−2.05
4986.90	915–20961	.024	.0089	−2.05
5008.22	1749–21710	.053	.020	−1.70
5047.41	2295–22101	.020	.0076	−2.12
5077.82	289–19977	.014	.0055	−2.26
5085.86	915–20571	.021	.0081	−2.09
5117.25	2294–21831	.041	.016	−1.80
5137.05	6445–25906	.031	.012	−1.92
5145.08	6283–25714	.035	.014	−1.85
5154.04	2294–21691	.022	.0086	−2.06
5160.33	5791–25163	.075	.030	−1.52
5184.59	5402–24684	.055	.022	−1.66
5204.32	6283–25492	.054	.022	−1.66
5225.12	5790–24923	.032	.013	−1.89
5238.61	9553–28636	.048	.020	−1.71
5247.35	5402–24453	.025	.010	−1.99
5247.75	4585–23635	.041	.017	−1.77
5257.04	5667–24684	.048	.020	−1.70
5278.18	5402–24342	.029	.012	−1.91
5288.40	5402–24305	.025	.010	−1.99
5310.04	0–18827	.012	.0050	−2.30
5311.88	4421–23241	.036	.015	−1.82
5312.73	5791–24608	.024	.010	−1.99
5321.60	5667–24453	.023	.010	−2.00
5327.76	6283–25047	.044	.019	−1.73
5349.92	4420–23107	.023	.0099	−2.01
5362.40	9690–28333	.047	.020	−1.70
5363.82	5667–24305	.025	.011	−1.97
5368.43	9075–27698	.057	.025	−1.61
5373.45	1749–20353	.0098	.0042	−2.37
5386.19	15392–33953	.24	.10	−.99
5403.20	5790–24293	.030	.013	−1.89
5405.98	8394–26887	.044	.019	−1.71
5444.48	5791–24152	.039	.017	−1.76
5465.68	9626–27917	.070	.031	−1.50
5475.72	9241–27499	.11	.052	−1.29
5480.27	12513–30756	.17	.078	−1.11
5481.21	6445–24684	.068	.031	−1.51
5482.54	15392–33627	.20	.091	−1.04
5434.55	1749–19977	.0094	.0042	−2.37
5487.00	5791–24010	.038	.017	−1.76
5491.22	8510–26716	.058	.026	−1.58
5492.97	0–18200	.044	.020	−1.70
5494.66	4420–22615	.027	.012	−1.92
5501.49	9553–27725	.040	.018	−1.74
5504.13	6445–24608	.046	.021	−1.68
5527.83	12513–30599	.17	.078	−1.11
5535.78	2294–20353	.013	.0059	−2.23
5538.53	9075–27126	.047	.022	−1.66
5544.81	4585–22615	.027	.012	−1.91
5548.05	12513–30533	.063	.029	−1.54
5551.42	6445–24453	.036	.017	−1.78
5552.60	6283–24288	.026	.012	−1.92
5570.66	9553–27499	.073	.034	−1.47
5580.81	5402–23315	.025	.012	−1.94
5581.23	914–18827	.015	.0070	−2.15
5581.61	289–18200	.019	.0088	−2.06
5587.17	8521–26415	.026	.012	−1.92
5597.37	6445–24305	.035	.017	−1.78
5602.90	6445–24288	.031	.014	−1.84
5603.97	5401–23241	.019	.0088	−2.06
5628.02	5791–23553	.020	.0094	−2.03
5638.00	8394–26126	.029	.014	−1.85
5644.21	12629–30341	.042	.020	−1.70
5653.77	2294–19977	.016	.0076	−2.12
5654.39	4421–22101	.022	.011	−1.97
5664.86	5667–23315	.019	.0092	−2.04
5674.88	9882–27499	.027	.013	−1.88
5683.33	9553–27143	.041	.020	−1.70
5691.40	6445–24010	.044	.021	−1.67
5704.07	8379–25906	.040	.020	−1.71
5706.99	9626–27143	.046	.022	−1.65
5723.63	5401–22868	.037	.018	−1.74
5733.21	8276–25714	.039	.019	−1.71
5743.10	0–17392	.010	.0051	−2.30
5743.44	8276–25667	.024	.012	−1.93
5776.87	9626–26931	.029	.015	−1.84
5788.59	6283–23553	.028	.014	−1.85
5791.74	9626–26887	.043	.024	−1.62
5798.54	5401–22642	.052	.026	−1.58
5811.27	8510–25714	.037	.019	−1.72
5827.99	4706–21860	.019	.0095	−2.02
5832.37	9882–27023	.041	.021	−1.68
5837.70	4585–21710	.039	.020	−1.70
5839.04	7166–24288	.019	.010	−2.00
5841.82	8379–25492	.033	.017	−1.78
5843.29	6445–23553	.029	.015	−1.83
5843.82	11787–28894	.040	.020	−1.69
5845.25	289–17392	.015	.0075	−2.12
5853.91	1749–18827	.016	.0081	−2.09
5859.16	13015–30078	.043	.025	−1.61
5870.93	5402–22429	.032	.017	−1.78
5886.93	8510–25492	.024	.012	−1.91
5895.32	6283–23241	.028	.014	−1.84
5932.44	5791–22642	.020	.010	−1.98
5934.46	8510–25356	.022	.012	−1.94
5952.05	6445–23241	.026	.014	−1.86
6004.83	9933–26581	.044	.024	−1.62
6017.39	4706–21320	.024	.013	−1.89
6051.74	914–17434	.021	.011	−1.94
6059.73	5667–22165	.020	.011	−1.95
6067.23	914–17392	.018	.010	−2.00
6087.34	6445–22868	.031	.017	−1.77
6110.91	2294–18654	.0074	.0042	−2.38
6164.77	10198–26415	.037	.021	−1.68
6181.37	8510–24684	.018	.010	−1.99
6254.22	6445–22429	.020	.012	−1.93
6279.64	5791–21710	.014	.0083	−2.08
6280.20	5402–21320	.025	.015	−1.83
6291.48	914–16804	.0049	.0029	−2.54
6322.37	0–15812	.0067	.0040	−2.40
6330.77	914–16706	.0057	.0034	−2.46
6336.55	8510–24288	.016	.0094	−2.03
6346.27	5401–21154	.0096	.0058	−2.24
6374.78	10285–25967	.028	.017	−1.77
6375.98	0–15679	.0037	.0023	−2.64
6378.52	289–15962	.011	.0067	−2.17
6379.64	5716–21387	.022	.014	−1.86
6386.84	5667–21320	.013	.0082	−2.08
6391.32	8510–24152	.014	.0085	−2.07
6400.36	5402–21021	.010	.0064	−2.20
6424.89	5402–20961	.013	.0079	−2.10
6448.04	9933–25437	.015	.0094	−2.03
6470.55	10740–26191	.017	.011	−1.97
6485.38	6445–21860	.0087	.0055	−2.26
6495.23	4585–19977	.0065	.0041	−2.38
6534.60	18654–33953	.057	.036	−1.44
6535.46	914–16211	.0062	.0040	−2.40
6536.58	5667–20961	.0096	.0061	−2.21
6549.88	7166–22429	.012	.0078	−2.11
6557.58	10740–25986	.021	.013	−1.87
6587.83	5526–20702	.0093	.0060	−2.22
6590.05	5402–20571	.0082	.0053	−2.27
6603.34	2294–17434	.0032	.0021	−2.68
6621.77	2294–17392	.0098	.0064	−2.19
6622.82	7547–22642	.013	.0083	−2.08
6640.50	9553–24608	.0097	.0064	−2.19
6671.21	8276–23262	.0079	.0053	−2.28
6676.92	18654–33627	.082	.055	−1.26
6701.68	9690–24608	.012	.0080	−2.10
6707.59	5667–20571	.0074	.0050	−2.30
6710.57	914–15812	.0035	.0024	−2.62
6717.45	7547–22429	.014	.0092	−2.04
6720.76	6445–21320	.0083	.0056	−2.25
6726.89	8379–23241	.011	.0076	−2.12
6742.47	5526–20353	.0051	.0035	−2.46
6771.03	914–15679	.0030	.0021	−2.68
6776.89	10740–25492	.016	.011	−1.96
6796.43	6445–21154	.0058	.0040	−2.40
6808.76	8423–23106	.0078	.0055	−2.26
6876.75	8379–22917	.041	.029	−1.54
6948.58	16211–30599	.061	.045	−1.35
6987.72	10740–25047	.015	.011	−1.95
7020.71	10444–24684	.029	.022	−1.66
7073.61	4663–18797	.0059	.0044	−2.35
7082.11	5401–19517	.011	.0085	−2.07
7183.47	2294–16211	.0060	.0046	−2.33
7218.04	5667–19517	.0085	.0066	−2.18
7379.63	10740–24288	.029	.024	−1.63
7454.03	10740–24152	.024	.020	−1.69
7510.08	8379–21691	.0098	.0083	−2.08
7580.91	7166–20353	.010	.0087	−2.06
7668.73	5790–18827	.0055	.0049	−2.31
7669.69	9882–22917	.010	.0092	−2.04
7802.40	10740–23553	.017	.016	−1.81
7837.71	9075–21831	.016	.014	−1.84
7976.88	5667–18200	.0091	.0086	−2.06
8040.10	8423–20857	.0065	.0063	−2.20
8074.03	6445–18827	.010	.0099	−2.01
8188.20	6445–18654	.010	.010	−2.00
8210.27	10740–22917	.015	.015	−1.83
8307.56	1749–13783	.0028	.0028	−2.55
8337.50	5401–17392	.012	.013	−1.89
8618.96	914–12513	.0023	.0026	−2.58
8702.08	2294–13783	.0034	.0038	−2.42
8787.37	4585–15962	.0065	.0075	−2.12

## Footnotes

1 Years in brackets indicate the literature reference at the end of this paper.

2 Bell and Upson [1971] investigated the intensity scale of Meggers, Corliss, and Scribner for the case of Fe I and concluded that the Fe I scale was too compressed. That conclusion does not support the present result.
